# Expanding the Molecular Spectrum of Papillary Renal Neoplasm With Reverse Polarity: A Report of Two Cases and a Literature Review

**DOI:** 10.7759/cureus.112738

**Published:** 2026-07-15

**Authors:** Gang Zhou, Xiuhua Dong, Lina Liu

**Affiliations:** 1 Pathology and Laboratory Medicine, Baylor Scott & White Medical Center - Temple, Temple, USA

**Keywords:** genitourinary pathology, kras mutations, next-generation sequencing, renal cancer, surgical pathology, urology

## Abstract

Papillary renal neoplasm with reverse polarity (PRNRP) is a rare renal tumor with distinct histomorphologic features and a strong association with KRAS exon 2 mutations, typically at codon 12. We report two cases that expand the molecular spectrum of this entity. The first tumor, a 3.5 cm mass in a 51-year-old female, harbored the classic KRAS codon 12 mutation (p.G12V), whereas the second, a 2.0 cm mass in a 77-year-old female, exhibited a KRAS p.Q61K mutation, rarely reported in PRNRP. Both tumors showed characteristic histology (tubulopapillary architecture, eosinophilic cytoplasm, apical nuclear polarity) and immunoprofile (GATA3+, CK7+, SDHB-retained, FH-retained). Next-generation sequencing using a 505-gene cancer panel also detected additional alterations in FLT3, POLD1, KMT2C, and WHSC1, the significance of which remains uncertain currently but may emerge with more molecular data from additional PRNRP cases. These findings underscore the value of integrated histopathologic and molecular evaluation, highlight KRAS Q61K as a rare variant, and provide a foundation for future genomic studies in this tumor type.

## Introduction

Papillary renal cell carcinoma (PRCC) is the second most common subtype of renal cell carcinoma, traditionally classified into prognostically distinct type one and type two categories [1,2]. The taxonomic understanding of PRCC has evolved significantly, beginning with reports describing variants with indolent behavior and oncocytic cells, termed "oncocytic PRCC" [3,4]. Subsequent refinement led to a proposal for a "type 4/oncocytic low-grade" subtype, notable for its GATA-3 positivity, CAIX negativity, and excellent prognosis [5].

This work culminated in the formal definition of papillary renal neoplasm with reverse polarity (PRNRP) as a distinct entity [6], a classification now formalized in the 2022 World Health Organization (WHO) system. PRNRP is characterized by a papillary and tubular architecture lined by a single layer of cells with eosinophilic cytoplasm and the unique histological hallmark of nuclei with "reverse polarity (apical positioning of the nuclei away from the basement membrane)." Immunohistochemically, routinely used to distinguish PRNRP from other renal neoplasms, it is consistently positive for GATA3 and CK7 (and often L1CAM) while showing retained expression of succinate dehydrogenase subunit B (SDHB) and fumarate hydratase (FH) [6,7]. Genomically, recurrent activating KRAS mutations are a defining feature, found in most cases and identified as an early driver event in its tumorigenesis [7,8].

Herein, we present two new cases of PRNRP and report a rare KRAS mutation p.Q61K. We highlight the diagnostic process and underscore the vital role of next-generation sequencing (NGS) not only in confirming the diagnosis but also in revealing a wider array of genetic alterations than previously characterized in this emerging entity.

## Case presentation

Case 1

A 51-year-old woman with no significant past medical history and no family history of renal or bladder cancer presented for evaluation of a right renal mass. The mass was discovered incidentally on a CT abdomen and pelvis performed for right lower quadrant abdominal pain. The initial CT (Figure 1A), followed by a dedicated MRI (Figure 1B) of the kidney, identified a 3.5 cm solid, suspicious mass in the mid portion of the right kidney. The patient denied any gross hematuria, history of kidney stones, or recurrent urinary tract infections. Her abdominal pain persisted but was not associated with other genitourinary symptoms.

**Figure 1:**
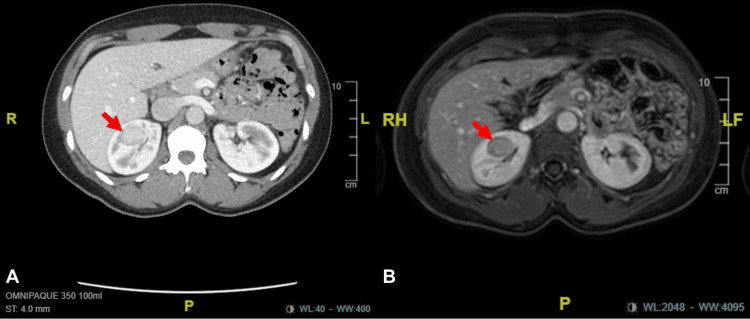
Pre-operative imaging identifies a right renal mass. (A) CT and (B) MRI (axial views) demonstrating a well-circumscribed 3.5 cm mass (arrows).

She subsequently underwent an uncomplicated partial nephrectomy. The resected specimen revealed a well-circumscribed 3.5 cm tumor. Histopathological examination demonstrated a neoplasm with a mixed tubular and papillary architecture. The neoplastic cells featured abundant eosinophilic cytoplasm and low-grade nuclear features. A critical diagnostic finding was the prominent apical positioning of the nuclei, away from the basement membrane-the hallmark of reverse polarity (Figures 2A-2B).

**Figure 2:**
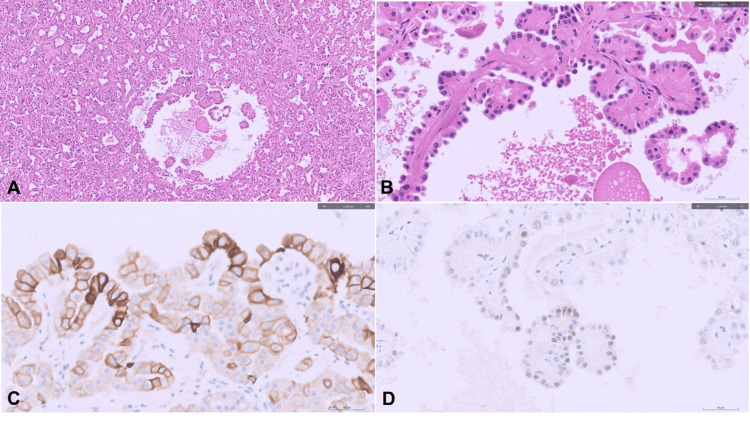
Pathological diagnosis of papillary renal neoplasm with reverse polarity (PRNRP). (A) Papillary architecture (H&E). (B) High-power view showing cells with eosinophilic cytoplasm and apically located nuclei (reverse polarity; H&E). (C) Positive CK7 (cytoplasmic) and (D) weak GATA3 (nuclear) immunohistochemical staining.

Immunohistochemical (IHC) staining was performed, revealing a profile positive for CK7 (Figure 2C) and GATA3 (Figure 2D), and negative for CK20, vimentin, CD117, and α-methylacyl-CoA racemase (AMACR). Retained expression of SDHB and FH ruled out succinate dehydrogenase-deficient and fumarate hydratase-deficient related renal carcinomas. The combined morphological and IHC findings supported a diagnosis of PRNRP, which was confirmed by expert genitourinary pathology consultation.

To corroborate the diagnosis, NGS was performed. This identified a pathogenic KRAS p.G12V missense mutation with a mutant allele fraction of 18.5%, providing definitive molecular confirmation of PRNRP. The analysis also revealed additional genetic alterations of potential clinical significance in FLT3 (p.N278T), POLD1 (p.R306C), PTPRT (p.M1237T), and TSC2 (p.S1487C) (Table 1). The tumor was microsatellite stable (MSS) and exhibited a tumor mutational burden (TMB) of 5.4 mutations per megabase, which is considered low.

**Table 1:** Comparative molecular profile of PRNRP. FLT3: FMS-like tyrosine kinase 3; CK7: cytokeratin 7; CK20: cytokeratin 20; KMT2C: lysine methyltransferase 2C (also known as MLL3); KRAS: Kirsten rat sarcoma viral oncogene homolog; MAF: mutant allele fraction; MSS: microsatellite stable; MSI: microsatellite instability; MSI-H: microsatellite instability-high; POLD1: DNA polymerase delta 1, catalytic subunit; PRNRP: papillary renal neoplasm with reverse polarity; PTPRT: protein tyrosine phosphatase receptor type T; pTNM: pathological tumor, node, metastasis staging system; SDH: succinate dehydrogenase; SDHB: succinate dehydrogenase complex iron sulfur subunit B; SGK1: serum/glucocorticoid-regulated kinase 1; TMB: tumor mutational burden; TSC2: tuberous sclerosis complex 2; WHSC1: Wolf-Hirschhorn syndrome candidate 1 (also known as NSD2); RCC: renal cell carcinoma

Gene	Alteration in Our Cases	Biological Context	Status in PRNRP	Tier
KRAS	p.G12V (Case 1); p.Q61K (Case 2)	Oncogenic driver; MAPK pathway activation.	Well-established, defining alteration. Mutually exclusive with BRAF mutations. Reported in ~80-90% of cases [9].	1B
FLT3	p.N278T (Case 1)	Receptor tyrosine kinase involved in proliferation and survival.	Not previously reported. Potential novel cooperating event.	III
POLD1	p.R306C (Case 1)	Catalytic subunit of DNA polymerase delta; critical for DNA replication and repair. Mutations can impair proofreading, leading to hypermutation.	Not previously reported. Of note, our cases had low TMB, suggesting this variant may not be functionally impairing in this context.	III
PTPRT	p.M1237T (Case 1)	Receptor-type tyrosine-protein phosphatase; putative tumor suppressor.	Not previously reported.	III
TSC2	p.S1487C (Case 1)	Negative regulator of the mTORC1 pathway.	Not previously reported. Inactivation is a hallmark of PEComas and other RCC subtypes.	III
KMT2C (MLL3)	p.P673R (Case 2)	Histone methyltransferase regulates chromatin remodeling and gene expression (tumor suppressor).	Not previously reported in PRNRP. Recurrently mutated in other cancers (e.g., urothelial carcinoma).	III
SGK1	p.N139S (Case 2)	Serine/threonine kinase regulates cell survival and ion transport.	Not previously reported.	III
WHSC1 (NSD2)	p. E1344Rfs*2 (Case 2)	Histone methyltransferase involved in epigenetic regulation (oncogenic).	Not previously reported.	III

Case 2

A 77-year-old female with a past medical history of cholelithiasis was found to have an incidental renal mass. The patient underwent a CT abdomen (Figure 3A) with renal mass protocol for an unrelated indication. The study revealed a 2.0 cm heterogeneously enhancing mass within the posterior mid left kidney, which was deemed suspicious for renal cell carcinoma. This finding was confirmed on a subsequent multiphase MRI (Figure 3B), which characterized the lesion as a 1.7 cm heterogeneously and mildly enhancing mass. Multiple simple cysts were noted in both kidneys.

**Figure 3:**
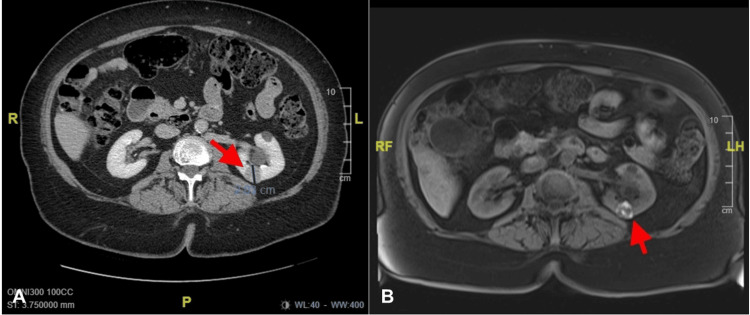
Imaging characteristics of the left renal mass. (A) Contrast-enhanced CT scan (axial view) and (B) T1-weighted MRI (axial view) demonstrating a well-circumscribed, heterogeneously enhancing mass (arrows) in the posterior mid left kidney.

A percutaneous biopsy of the left renal mass was performed. Histological examination revealed a low-grade papillary neoplasm comprised of cells with eosinophilic cytoplasm and suggestive nuclear features (Figures 4A-4B). IHC staining was pivotal, demonstrating a profile positive for CK7 (Figure 4C), EMA, Pax-8, and GATA3 (Figure 4D), with focal positivity for vimentin and CD10. The tumor was negative for CK20, CD117, CAIX, and AMACR. Based on the morphological and IHC findings, a diagnosis of "PRNRP is favored" was rendered.

**Figure 4:**
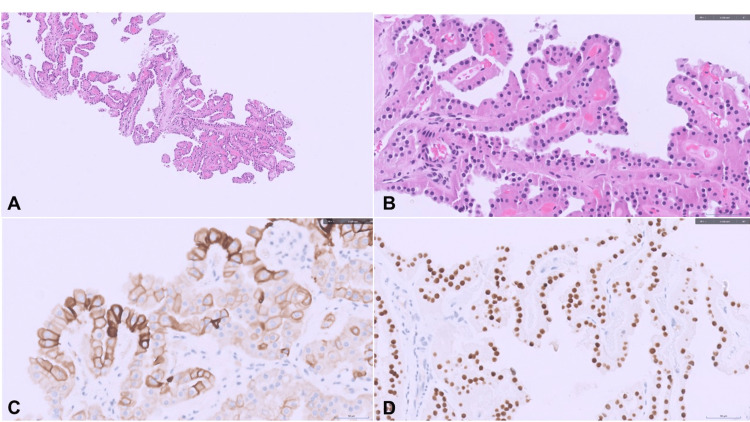
Histopathologic and immunohistochemical features diagnostic of papillary renal neoplasm with reverse polarity (PRNRP). (A) Low-power view shows a predominant papillary architecture with fibrovascular cores (H&E stain). (B) High-power view reveals the hallmark cytologic finding: neoplastic cells with abundant eosinophilic cytoplasm and low-grade nuclei exhibiting reverse polarity (H&E stain). (C) Positive CK7 (cytoplasmic) and (D) strong GATA3 (nuclear) immunohistochemical staining.

To confirm the diagnosis, NGS was performed on the biopsy specimen. This analysis identified a pathogenic KRAS p.Q61K missense mutation with a high mutant allele fraction of 32.1%, providing definitive molecular confirmation of PRNRP. The molecular profile also revealed additional variants of uncertain significance in KMT2C (p.P673R), SGK1 (p.N139S), and WHSC1 (p.E1344Rfs2) (Table 1). Notably, no pathogenic alterations were found in classic renal cell carcinoma genes such as VHL or MET, and no gene fusions were detected. The tumor was MSS and exhibited a low TMB of 4.6 mutations per megabase.

## Discussion

PRNRP is a recently defined renal tumor entity, previously classified as a subtype of PRCC. It is now recognized as a distinct clinicopathologic entity with characteristic morphological, IHC, and molecular features [1,3,4]. The diagnosis in our two cases was established through the classic triad of histology, IHC, and molecular confirmation, beautifully illustrating the modern diagnostic approach to renal neoplasms.

Histologically, PRNRP is characterized by papillary or tubular structures lined by a single layer of oncocytic cells with abundant eosinophilic cytoplasm [9]. The pathognomonic feature is the presence of low-grade nuclei positioned apically away from the basement membrane, known as reverse polarity [10]. Immunohistochemically, these tumors are typically positive for CK7, GATA3, and L1CAM, while being negative for vimentin and AMACR [11]. While our first case exhibited this classic IHC profile, the second case showed focal vimentin positivity, a finding that, while uncommon, has been reported and underscores the importance of interpreting IHC within the full diagnostic context [11]. Genetically, PRNRP is defined by a high frequency of recurrent KRAS missense mutations, reported in approximately 84% of cases [10,12,13]. This stands in stark contrast to other RCC subtypes, where KRAS mutations are exceedingly rare (e.g., 0.4% in clear cell RCC, 1.8% in other PRCC, and 0% in chromophobe RCC) [5,12,14].

Both our patients were adult females (aged 51 and 77) who presented with small, incidentally discovered renal masses (3.5 cm and 2.0 cm), consistent with the reported demographics and clinical presentation of PRNRP [1,12]. Histological examination of both tumors revealed the hallmark features of eosinophilic cells with low-grade nuclei demonstrating reverse polarity. The diagnosis was solidified by IHC, showing positivity for CK7 and GATA3, and further confirmed molecularly by NGS, which identified pathogenic KRAS mutations - p.G12V in Case 1 and p.Q61K in Case 2. The p.G12V mutation is the most commonly reported variant in PRNRP, accounting for over half of all cases [7,8,13]. The KRAS p.Q61K mutation has been reported in one prior PRNRP case [15], and our case adds to the limited literature. This variant is a well-established pathogenic KRAS mutation in non-small cell lung carcinoma and provides strong molecular support for the diagnosis.

Beyond confirming the diagnosis, our cases contribute novel genetic findings to the PRNRP landscape. Both tumors were MSS and had a low TMB, features aligning with the indolent behavior of this entity. However, NGS also revealed secondary genetic alterations of potential significance. Case 1 harbored variants in FLT3, POLD1, PTPRT, and TSC2, while Case 2 had variants in KMT2C, SGK1, and WHSC1. The co-occurrence of these alterations, particularly in genes involved in tyrosine kinase signaling (FLT3), DNA replication fidelity (POLD1), and chromatin remodeling (KMT2C, WHSC1), suggests that PRNRP oncogenesis may involve cooperative genetic events beyond the initiating KRAS driver. The functional and prognostic implications of these variants are currently unknown and warrant further investigation in larger cohorts.

The consistent presence of KRAS mutations in PRNRP places it within a broader family of KRAS-driven neoplasms, which includes adenocarcinomas of the lung, colon, and pancreas [16,17]. The KRAS protein is a critical regulator of the MAPK/ERK, PI3K/AKT, and other signaling pathways that promote cellular proliferation and survival [18,19]. Interestingly, activating KRAS mutations are also found in several benign or low-grade papillary lesions, such as oncocytic sinonasal papillomas, suggesting that the biological outcome of KRAS activation is highly context-dependent [20]. In PRNRP, the mutation appears to drive a neoplasm with a very indolent clinical course. The prognosis for patients with PRNRP is excellent, with no reported cases of disease progression or recurrence in the literature to date, including follow-up extending nearly 20 years [1,3,4]. The mainstay of treatment is surgical resection, typically partial nephrectomy, as performed in our first case.

Moving forward, a better definition of these genotype-phenotype correlations and their impact on long-term patient outcomes will be crucial for refining prognostic stratification, guiding therapeutic decisions, and optimizing genetic counseling and patient care.

## Conclusions

We present two classic cases of PRNRP where integrated histopathological and molecular analysis confirmed the diagnosis. The discovery of uncommon mutations expands the known genetic spectrum of this entity and opens new avenues for research into its biology. These cases underscore the importance of combining traditional microscopic evaluation with modern molecular techniques for the accurate diagnosis and future understanding of emerging renal tumor entities.
